# Evaluation of 18 S rRNA as an endogenous control in OpenArray^®^ plasma RNA qPCR workflows

**DOI:** 10.1007/s11033-026-12820-9

**Published:** 2026-09-27

**Authors:** Habib Francis, Andrzej S. Januszewski, Abubakar Mangani, Fahmida K. Ema, Anandwardhan A. Hardikar, Mugdha V. Joglekar, Michael L.H. Huang, David R. Sullivan, Ronald C.W. Ma, Sanjeev Galande, Val Gebski, R John Simes, Alicia J. Jenkins, Mark P. Molloy, Anthony C. Keech

**Affiliations:** 1 Sydney Clinical Trials Centre, The University of Sydney, Sydney, Australia; 2 Sydney Pharmacy School, The University of Sydney, Sydney, Australia; 3 Western Sydney University School of Medicine, Campbelltown, Australia; 4 Baker Heart and Diabetes Institute, Melbourne, Australia; 5 Royal Prince Alfred Hospital, Camperdown, Australia; 6 Department of Medicine and Therapeutics and Li Ka Shing Institute of Health Sciences, Faculty of Medicine, Prince of Wales Hospital, The Chinese University of Hong Kong, Hong Kong, China; 7 Shiv Nadar Institution of Eminence, Tehsil Dadri, Gautam Buddha Nagar, Uttar Pradesh India; 8 Bowel Cancer and Biomarker Research Laboratory, School of Medical Sciences, Kolling Institute, The University of Sydney, St Leonards, Australia

**Keywords:** qPCR, OpenArray, 18S rRNA, Pre-amplification, Plasma RNA, Endogenous control, Gene expression

## Abstract

**Background:**

Reliable qPCR analysis depends on stable endogenous controls, particularly in high-throughput pre-amplification workflows where technical bias may arise. The suitability of highly abundant 18 S rRNA as a reference gene in multiplex OpenArray^®^ platforms remains debated. This study evaluated the stability of 18 S rRNA, its impact on co-amplified targets, and comparability between archived and newly manufactured primer pools.

**Methods:**

Plasma RNA from the Fenofibrate Intervention and Event Lowering in Diabetes (FIELD) trial was analysed using TaqMan OpenArray^®^ following reverse transcription and pre-amplification. Paired samples (*n* = 23) were pre-amplified with primer pools with and without 18 S rRNA and compared across four target genes. Stability of 18 S rRNA was assessed in 789 samples across 17 runs. Agreement between archived and new primer pools was evaluated in 774 paired samples using Wilcoxon signed-rank and Bland–Altman analyses.

**Results:**

18 S rRNA showed stable detection (median Ct 14.5, IQR 13.4–16.3) with low inter-run variability (CV 3.9%). Inclusion of 18 S rRNA during pre-amplification did not significantly alter Ct values of co-amplified targets (median differences 0.07–0.52 cycles; *p* > 0.05), with minimal overall bias (-0.18 Ct). Archived and newly manufactured primer pools showed a small but significant Ct shift, with lower values in the new pool (bias 0.53 Ct; *p* < 0.0001), although variability between paired measurements was wide.

**Conclusion:**

18 S rRNA is a stable technical control in plasma OpenArray^®^ workflows and did not measurably bias co-amplified targets during pre-amplification. Archived and new primer pools showed only minor systematic differences and remained broadly comparable at the group level.

## Highlights

18 S rRNA showed consistent detection across plasma OpenArray workflows.

18 S inclusion did not alter Ct values of co-amplified targets.

Archived and new primer pools showed only modest Ct differences.

Plasma-derived 18 S rRNA performance was systematically evaluated.

Findings support the technical utility of 18 S rRNA within the evaluated plasma OpenArray workflow.

## Introduction

Accurate gene expression measurement by quantitative real-time PCR (qPCR) depends on effective normalisation using a suitable and stable endogenous gene targets (endogenous controls) that are invariably expressed in different biological samples, in order to account for potential technical variation introduced during RNA extraction, reverse transcription, amplification, and reaction preparation. This issue is particularly relevant in high-throughput platforms such as the TaqMan OpenArray^®^ and other microfluidic systems, where limited reaction volumes and multiplexed workflows often require a pre-amplification step to increase cDNA input. Although pre-amplification improves sensitivity across multiple parallel targets, it may introduce bias when co-amplified transcripts differ substantially in abundance [1].

A commonly used endogenous control is 18 S ribosomal RNA (18 S rRNA) because of its high abundance and its frequent assumption of relative stability [2]. However, validation studies evaluating 18 S rRNA as a reference gene have produced mixed findings [3, 4], raising concerns about its suitability as a universal control across all experimental settings. In some animal models, 18 S rRNA has been reported to be among the least stable candidate reference genes, suggesting that its expression or detection may vary in some physiological or pathological conditions [4, 5]. In other settings, although 18 S rRNA performed well as a reference gene, its very high abundance required greater template dilution to bring it within the dynamic range of detection of lower-abundance targets [6].

The high abundance of 18 S rRNA has also raised concerns in multiplexed pre-amplification settings [1, 7]. Highly abundant transcripts may reach detectable thresholds earlier and could theoretically consume shared reagents disproportionately during amplification, potentially affecting the amplification efficiency or sensitivity of lower-abundance targets. Several studies have therefore suggested that inclusion of highly abundant targets in pre-amplification pools should be considered carefully to minimise potential bias [7, 8]. These concerns are particularly relevant in high-throughput workflows, where many targets are co-amplified and small differences in efficiency may be magnified.

In this short report, we describe experiments designed to address two related questions in the context of the TaqMan OpenArray^®^ plasma RNA workflows: (1) whether inclusion of the 18 S rRNA assay in the pre-amplification pool alters cycle threshold (Ct) values of co-amplified target genes; and (2) whether 18 S rRNA measurements obtained using an archived pool with 18 S rRNA are comparable to those obtained using a newly manufactured pool with 18 S rRNA.

## Methods

### Samples

All sodium-citrate plasma samples used in this analysis were obtained from the Fenofibrate Intervention and Event Lowering in Diabetes (FIELD) trial of adults with Type 2 diabetes [9]. All participants provided written informed consent. The study protocol was approved by the Human Research Ethics Committee of The University of Sydney and national ethics committees (Finland and New Zealand) and was undertaken in accordance with the Declaration of Helsinki and Good Clinical Practice Guidelines.

The sample set included individuals with and without diabetes-related complications at baseline, individuals who developed complications during follow-up, and individuals who remained complication-free during trial follow-up. Plasma samples (3 freeze-thaw cycles) were assessed in separate analyses using different target pre-amplification primer pools, with partial overlap between panels enabling the comparisons described here.

To assess the performance and stability of 18 S, 789 samples were included in the analysis. To evaluate whether inclusion of 18 S rRNA in the pre-amplification pool affected amplification of other targets, 23 paired samples were analysed after pre-amplification using a pool with 18 S rRNA and a pool without 18 S rRNA. The effect of 18 S rRNA inclusion was assessed by comparing expression of Actin gamma 1 (ACTG1), Actin beta (ACTB), Clusterin (CLU) and Profilin 1 (PFN1) genes in the same samples under the two pre-amplification conditions. The corresponding TaqMan assay identifiers used for OpenArray were [ACTG1: Hs03044422_g1], [ACTB: Hs01060665_g1], [CLU: Hs00156548_m1], [PFN1: Hs00748915_s1] and [18 S: Hs99999901_s1], respectively. Because not all 23 samples had detectable levels of all target genes, the effective sample sizes differed by assay: ACTG1 (*n* = 17), ACTB (*n* = 17), CLU (*n* = 8), and PFN1 (*n* = 9). These 4 genes were selected as representative target genes because they were already included in the OpenArray target panel used in our previous FIELD study [10, 11], allowing assessment of whether inclusion of 18 S rRNA in the pre-amplification primer pool affected amplification of established study targets. For comparison of 18 S rRNA Ct values obtained under the two primer pool conditions, 18 S rRNA Ct values were analysed in 774 of the 789 samples.

### Plasma RNA extraction and reverse transcription

Total RNA was extracted from 200 µL sodium citrate plasma using the RNeasy 96 QIAcube HT Kit (QIAGEN, Hilden, Germany) according to the manufacturer’s instructions. Reverse transcription (RT) was performed using 50 ng RNA and the High-Capacity cDNA RT Kit (Thermo Fisher Scientific, Waltham, MA).

The cDNA was then pre-amplified using custom OpenArray^®^ PreAmp primer pools. Depending on the comparison being performed, samples were pre-amplified using either a newly manufactured primer pool with 18 S rRNA, a newly manufactured primer pool without 18 S rRNA, or an archived primer pool with 18 S rRNA that had been stored (-80 °C, never thawed) for more than 5 years. Pre-amplification was performed for 14 cycles on a ViiA 7 Real-Time PCR System (Thermo Fisher Scientific) before OpenArray^®^ qPCR analysis.

### TaqManOpenArray^®^ technology

All OpenArray^®^ assay materials, accessories, and reagents were obtained from Thermo Fisher Scientific (Waltham, MA) and all procedures were performed according to the manufacturer’s protocols. Briefly, pre-amplified cDNA was diluted 1:20, and 2.5 µL diluted cDNA was combined with 2.5 µL TaqMan OpenArray^®^ Real-Time PCR Master Mix in an OpenArray^®^ 384-well sample plate. The reaction mixture was then transferred to a TaqMan OpenArray^®^ Real-Time PCR Plate using the automated OpenArray^®^ AccuFill system. Plates were sealed and run on a QuantStudio 12 K Flex Real-Time PCR Instrument. Plate design, sample layout, and thermocycling conditions were uploaded electronically before each run. OpenArray^®^ data were analysed using QuantStudio™ 12 K Flex OpenArray^®^ Analysis Software (Thermo Fisher Scientific). Ct values were generated using automatic baseline and threshold settings. Quality control metrics, including amplification score and Ct confidence, were applied according to the manufacturer’s default criteria. Ct values were then exported to Microsoft Excel for downstream analysis.

### Statistical analysis

18 S rRNA detectability and variability were assessed using descriptive statistics, including mean ± standard error of the mean (SEM), median (lower quartile, upper quartile), and coefficient of variation (CV). Inter-run variability was assessed by comparing Ct values across runs. Ct values below 10 were excluded as they likely reflect signal saturation outside the assay’s reliable dynamic range.

Paired comparisons of Ct values between experimental conditions were performed using the Wilcoxon signed-rank test. Statistical analyses were performed using GraphPad Prism (Version 11.0.0; Dotmatics, California, USA), and a two-sided p-value < 0.05 was considered statistically significant. Agreement between conditions was assessed using Bland–Altman analysis.

## Results

### Stability of 18 S rRNA Across Plasma Samples

**Fig. 1:**
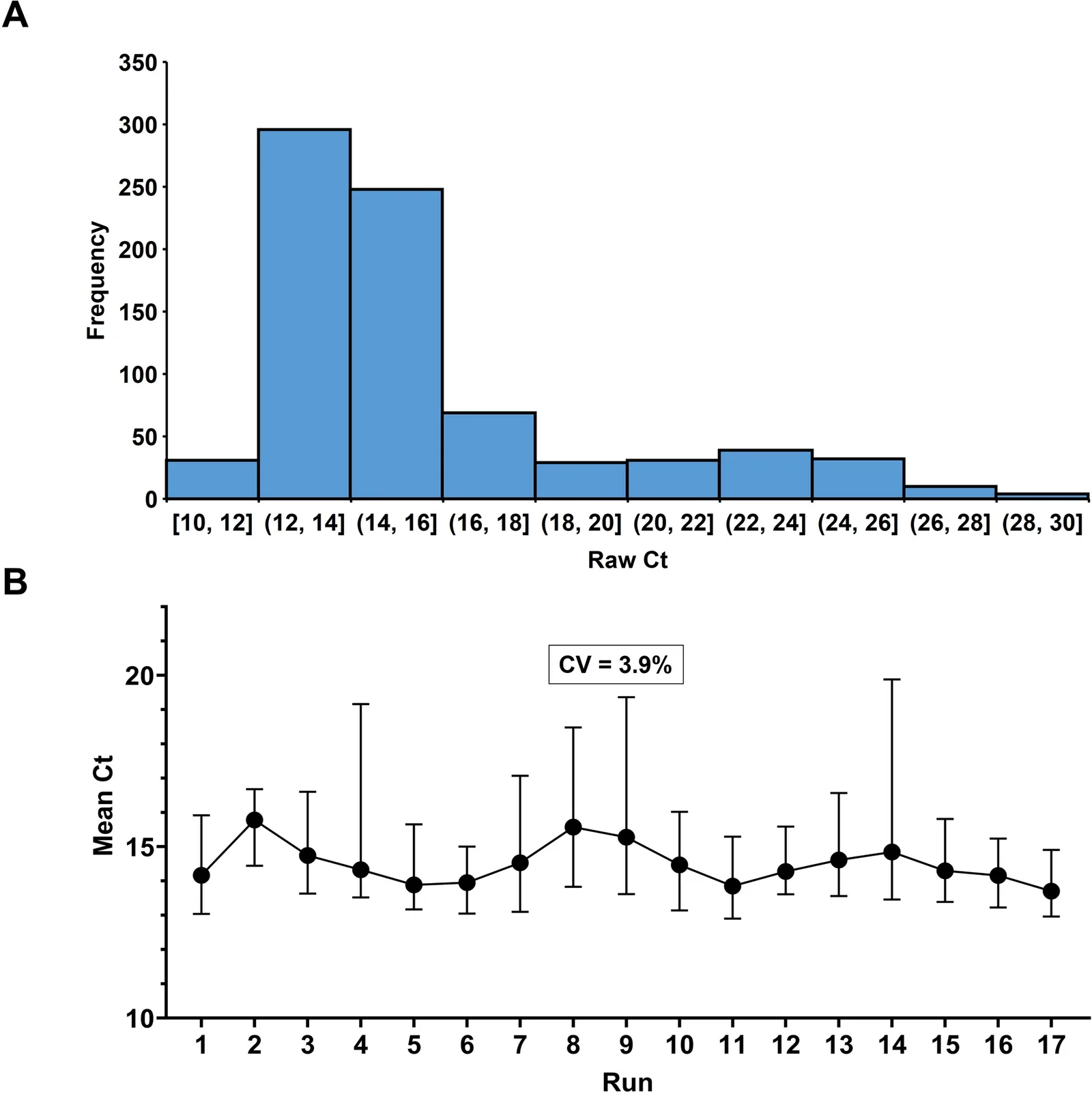
Detection and technical stability of 18 S rRNA in plasma samples. (A) Distribution of 18 S rRNA Ct values across plasma samples. (B) Inter-run variability of median (25th, 75th percentile) 18 S rRNA Ct values across runs

Across 17 runs, 18 S rRNA was consistently detectable across the analysed plasma samples with median Ct of 14.5 (IQR 13.4–16.3) and a mean Ct of 15.7 ± 0.13 (Fig. 1A). The distribution of Ct values was unimodal, with most measurements clustering around the median and no obvious evidence of bimodality or systematic shifts suggestive of batch effects or major technical artefacts. A small number of higher-Ct outliers were observed, but these did not dominate the overall distribution (Fig. 1A).

Assessment of inter-run performance showed closely aligned mean 18 S rRNA Ct values across runs, with an inter-run coefficient of variation of 3.9% and no significant differences between runs, indicating no systematic shift in Ct values across runs (Fig. 1B).

### Effect of inclusion of 18 S rRNA in the pre-amplification pool

**Fig. 2:**
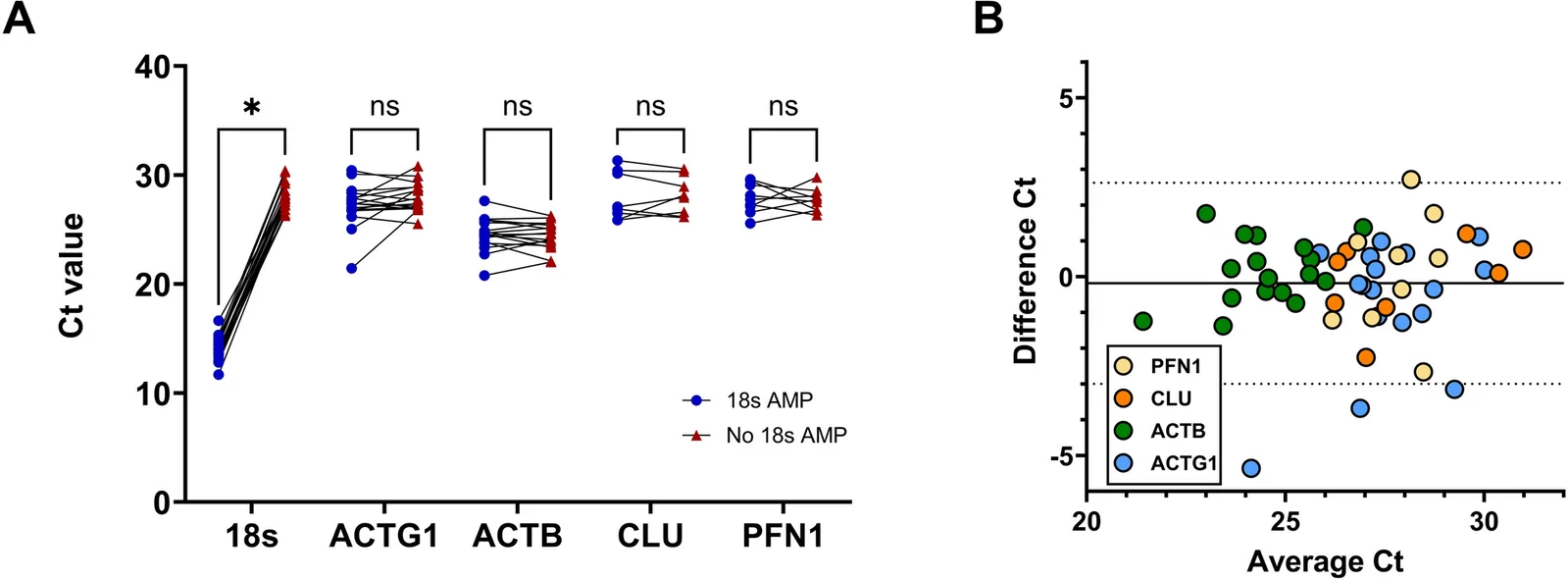
Effect of inclusion of 18 S rRNA in the pre-amplification pool on raw target gene Ct values. (A) Paired Ct values for ACTG1, ACTB, CLU, and PFN1 following pre-amplification using a pool with 18 S rRNA or a pool without 18 S rRNA. Lines connect paired measurements from the same sample. Statistical significance was assessed using Wilcoxon signed-rank test. (B) Bland–Altman plot showing the difference in Ct values between conditions plotted against the mean Ct for each paired sample. Each point represents an individual sample, coloured by target gene. The solid horizontal line indicates the mean difference (bias), and the dashed lines represent the 95% limits of agreement (mean ± 1.96 SD)

To assess whether inclusion of a highly abundant reference assay influenced amplification of co-amplified targets, paired Ct values were compared for four target genes (ACTG1, ACTB, CLU, and PFN1) in identical plasma RNA samples pre-amplified using two primer pools: a pool with 18 S rRNA and a pool without 18 S rRNA. As expected, the raw Ct values for 18 S rRNA are significantly higher (i.e. less abundant) in the samples without pre-amplification (Fig. 2A). Raw Ct values of the four target genes were similar between the two pre-amplification conditions (Fig. 2A). Median paired Ct differences between conditions ranged from 0.07 to 0.52 cycles, with no statistically significant differences detected for any of the four targets (*p* > 0.05; Fig. 2A).

Bland–Altman analysis across the four target genes demonstrated consistent agreement between pre-amplification conditions with and without 18 S rRNA primers (Fig. 2B). The mean difference was close to zero (-0.18) in the combined analysis of four gene targets, indicating minimal systematic bias. No gene-specific shift in bias was observed with data points for each gene similarly distributed and mostly falling within the limits of agreement. These findings further support that, for the targets examined, inclusion of 18 S rRNA in the pre-amplification pool was not associated with a consistent shift in raw Ct values or evidence of substantial systematic bias.

### Comparison of archived and newly prepared primer pools

To assess comparability between archived and newly generated data, 18 S rRNA Ct values were compared in 774 paired plasma samples amplified using either a newly manufactured primer pool with 18 S rRNA or an archived primer pool with 18 S rRNA. Amplification was successful under both conditions.

**Fig. 3:**
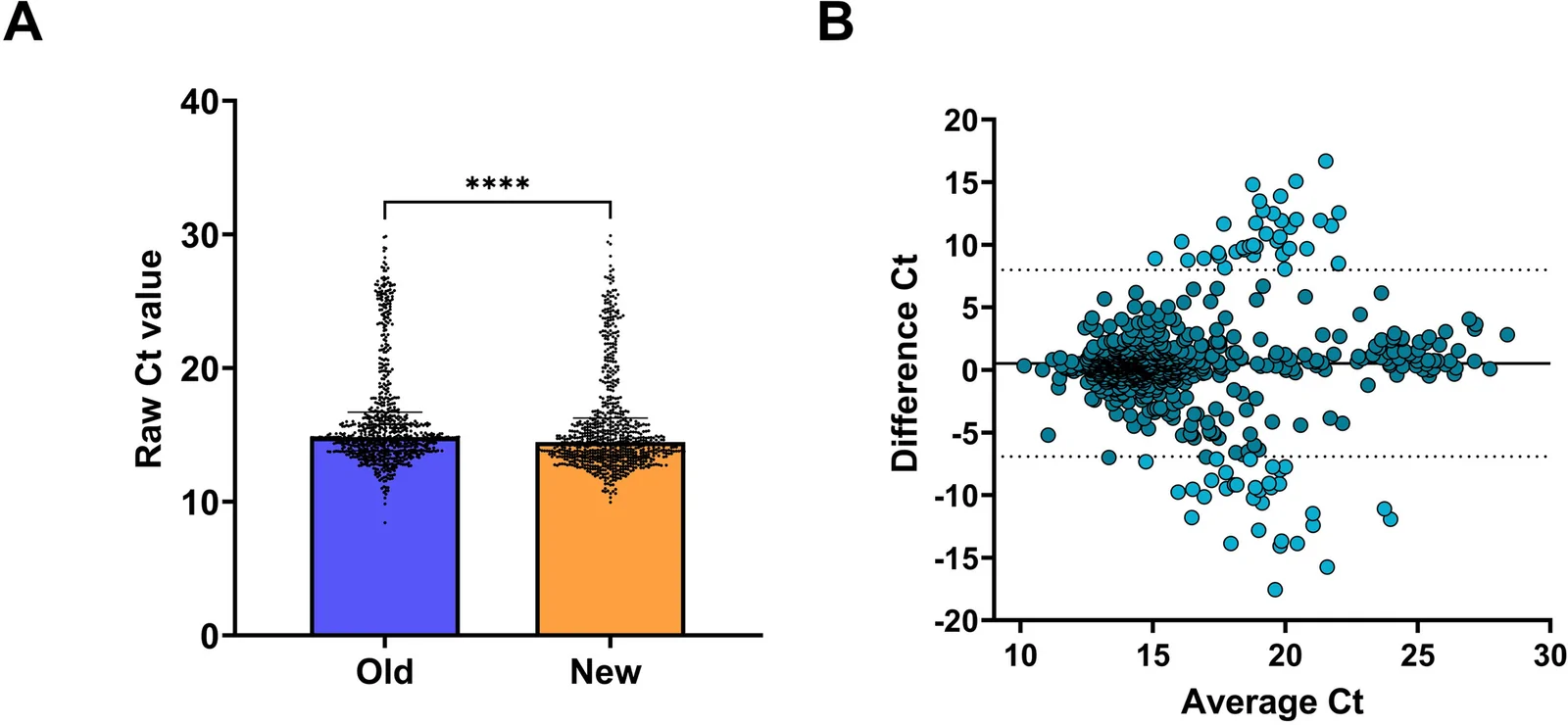
Comparison of 18 S rRNA Ct values obtained using archived and newly prepared pre-amplification pools. (A) Mean Ct values for 18 S rRNA obtained using archived and newly prepared pools; error bars represent standard deviation. Statistical significance was assessed using Wilcoxon signed-rank test. (B) Bland–Altman plot comparing Ct values obtained using the archived gene pool and the newly manufactured gene pool containing 18 S rRNA. The difference in Ct values between conditions is plotted against the mean Ct for each paired sample. Each point represents an individual sample. The solid horizontal line indicates the mean difference (bias), and the dashed lines represent the 95% limits of agreement (mean ± 1.96 SD). Dark blue dots indicate values within the 95% limits of agreement, whereas light blue dots indicate values outside the 95% limits of agreement

A small but consistent shift in Ct values was observed between the two primer pools, with the newly prepared pool having a slightly lower but significant median Ct of 14.5 (IQR 13.4–16.3; *p* < 0.0001) than the archived pool with median Ct of 14.8 (IQR 13.8–16.7) (Fig. 3A). Although this difference was statistically significant (Wilcoxon signed-rank test, *p* < 0.0001), the absolute magnitude of the shift was modest.

Bland–Altman analysis demonstrated a small mean bias of 0.53 Ct between archived and newly manufactured pools, indicating only a modest shift in 18 S rRNA amplification. However, variability between paired measurements was substantial (SD 3.82 Ct), with 95% limits of agreement ranging from − 6.95 to 8.01 Ct. Visual inspection of the Bland–Altman plot suggested increasing variability at higher average Ct values, consistent with a possible proportional effect, although no strong systematic trend was evident across the full measurement range. The wide limits of agreement indicate that the two conditions should be regarded as broadly comparable at the group level rather than directly interchangeable for individual paired measurements. (Fig. 3B).

## Discussion

### Technical performance of 18s

Numerous studies and guidelines have emphasised that reference genes should be empirically validated within the specific experimental context, rather than assumed to be stable across different sample types and workflows [12, 13]. In this study, 18 S rRNA showed consistent detection across a large set of plasma-derived RNA samples, with low inter-run variability and a unimodal distribution of Ct values. Importantly, these findings were obtained using archived plasma samples that had undergone multiple freeze–thaw cycles, suggesting that 18 S rRNA measurements may remain technically robust under conditions commonly encountered in longitudinal and clinical biobanking studies.

Although the low inter-run variability observed in Ct values supports the technical consistency of 18 S rRNA measurement in this workflow, Ct variability alone does not establish biological stability of an endogenous control across different experimental or biological conditions. The present study was designed to evaluate the technical performance of 18 S rRNA within the plasma OpenArray workflow rather than to comprehensively compare the biological stability of multiple candidate reference genes.

### Effect of 18s on co-amplified targets

The present findings suggest that, within this plasma-based OpenArray^®^ workflow, 18 S rRNA performed consistently as a technical control and was not associated with an obvious systematic shift in raw Ct values of the co-amplified targets examined during multiplex pre-amplification. This is important because pre-amplification has been recognised as a potential source of technical bias in RT-qPCR workflows, particularly when targets differ markedly in abundance [1, 14]. Given the high abundance of 18 S rRNA, concerns have been raised that its inclusion in multiplex pre-amplification reactions could adversely affect lower-abundance targets through competition for shared reagents. Our results do not support a major effect of this kind under the conditions tested, although this conclusion is based on a limited number of paired samples and only four targets, with small effective sample size for some targets.

### New vs. archived gene pools

The comparison between archived and newly manufactured primer pools addresses an additional practical issue relevant to long-term and longitudinal studies. Although a statistically significant difference in mean Ct values was observed between the two pools, the magnitude of this shift was modest and amplification remained successful under both conditions. The small average shift between paired measurements suggests that data generated using archived reagents may remain broadly interpretable at the group level, however, the wide variability between paired measurements indicates that direct comparison with newly generated data should be made cautiously. The observed variability cannot be attributed specifically to degradation of the archived primer pool, as the pool had remained unopened and stored at − 80 °C and no intermediate measurements were available to assess changes in performance over time. Differences between manufacturing lots, primer concentration or formulation, amplification efficiency, and sample-specific technical variability may all contribute to differences between primer pools. In addition, the plasma samples had undergone multiple freeze–thaw cycles, which may have contributed to measurement variability. The greater spread of differences at higher mean Ct values may also reflect increased measurement variability at lower template abundance.

It is also important to consider that these experiments were performed using RNA extracted from plasma, a biofluid characterised by low total mRNA content and a predominance of fragmented and extracellular RNA species [15]. In this setting, the relative abundance and integrity of 18 S rRNA and other endogenous control targets may differ substantially from those in cellular RNA preparations [16]. In addition, pre-analytical variables, including blood collection tube type and anticoagulant choice, have been shown to influence extracellular RNA profiles and may therefore affect assay performance and comparability across studies [17]. Accordingly, these findings should be interpreted within the context of plasma-based OpenArray^®^ workflows and should not be assumed to apply directly to other sample types or assay configurations.

### Experimental Suggestions

When replacement of a primer pool is required, retaining the primer sequences, assay specifications, primer concentrations and manufacturing/lot information from the original pool would facilitate comparison with the replacement pool. Although assay identifiers and target specifications for the assays used in this study are available from the manufacturer, the individual primer sequences are not disclosed. Consequently, sequence-level identity between the newly manufactured pool and the archived pool could not be confirmed retrospectively because the specifications of the archived pool at the time of manufacture were not available. Recording these details prospectively may therefore help identify potential changes between primer-pool lots and assist interpretation of longitudinal differences in assay performance.

For longitudinal studies, maintaining the same primer-pool lot across study time points may minimise the introduction of technical variability associated with primer-pool changes. When replacement of a primer pool is unavoidable, a bridging experiment using a representative subset of samples analysed with both the existing and newly manufactured pools could be performed before transitioning between pools. Paired comparisons and Bland–Altman analysis could be used to assess systematic bias and individual-level agreement. Where a small and consistent bias is identified, appropriate calibration or statistical adjustment may be possible. However, where substantial or inconsistent variability is observed, the two pools should not be assumed to be interchangeable based solely on the mean difference. In such circumstances, reanalysis using a common primer-pool lot, where feasible, or adoption of an alternative validated assay or platform measuring the same target may be considered. Inclusion of stable quality-control samples across experimental runs could provide additional monitoring of assay performance following a primer-pool transition. It is also important to consider whether pre-amplification is required throughout a longitudinal study. Where sufficient target signal can be obtained without pre-amplification, subsequent time points could potentially be analysed using a validated workflow without this additional amplification step, thereby avoiding variability associated with changes in pre-amplification primer pools.More broadly, the analytical approach applied here, combining assessment of signal stability, paired technical comparisons, and comparison of historical and newly generated data, may provide a practical framework for evaluating endogenous controls’ performance and data comparability in other high-throughput qPCR settings. As with any reference assay, however, suitability should be established within the specific platform, assay design, and sample type under study.

### Strengths and Limitations

A key strength of this study is the use of a large, well-characterised clinical cohort (FIELD trial) with a substantial number of plasma samples, enabling robust assessment of 18 S rRNA performance across multiple runs and experimental conditions. The study design incorporates both technical replication (paired pre-amplification conditions with and without 18 S rRNA) and real-world workflow evaluation, including comparison of archived and newly manufactured primer pools. The use of multiple complementary statistical approaches, including paired testing and Bland–Altman analysis, further strengthens the reliability and interpretability of the findings. Importantly, analyses were conducted within a clinically relevant plasma RNA OpenArray^®^ workflow, enhancing translational relevance for high-throughput biomarker studies.

Limitations include the relatively small number of paired samples available for assessing the impact of 18 S rRNA inclusion on co-amplified targets, with reduced effective sample sizes for some genes due to undetectable expression. Only four target genes were evaluated in this context, limiting generalisability across the full transcript panel. Additionally, while overall bias was minimal, the wide limits of agreement observed between archived and newly manufactured primer pools indicate variability at the individual sample level, which may limit interchangeability for longitudinal or paired analyses. Finally, findings are specific to plasma-derived RNA and the OpenArray^®^ platform and may not directly translate to other sample types, platforms, or pre-amplification chemistries.

## Conclusion

In summary, this study provides, to our knowledge, the first systematic evaluation of 18 S rRNA inclusion in multiplex pre-amplification within plasma-derived OpenArray workflows, alongside an assessment of archived versus newly manufactured primer pool comparability. Our findings support the technical utility of 18 S rRNA within plasma OpenArray qPCR workflows, with no consistent evidence of systematic bias in the raw Ct values of co-amplified targets. However, these findings do not establish the biological stability of 18 S rRNA as an endogenous control across different experimental or disease conditions, nor do they imply equivalence of 18 S rRNA-normalised expression values across conditions or for targets not examined here.

## Data Availability

Deidentified participant data and relevant study variables supporting the findings of this study may be made available upon reasonable request for academic use, subject to the limitations of the informed consent provided by FIELD participants and approval by representatives of the FIELD trial Steering Committee. Requests should be made to the corresponding author. Approved researchers will be required to sign a data access agreement with the National Health and Medical Research Council of Australia (NHMRC) Clinical Trials Centre of the University of Sydney. Because of participant consent and governance restrictions, the full dataset cannot be deposited in a public repository.
